# Artificial intelligence in otorhinolaryngology: current trends and application areas

**DOI:** 10.1007/s00405-025-09272-5

**Published:** 2025-02-17

**Authors:** Emre Demir, Burak Numan Uğurlu, Gülay Aktar Uğurlu, Gülçin Aydoğdu

**Affiliations:** 1 Department of Biostatistics, Faculty of Medicine, Hitit University, Çorum, Turkey; 2 Private Clinic, Otorhinolaryngology, Çorum, Turkey; 3 Department of Otorhinolaryngology, Faculty of Medicine, Hitit University, Çorum, Turkey

**Keywords:** Otorhinolaryngology, Artificial intelligence, AI, Deep learning, Bibliometric analysis, Research trends

## Abstract

**Purpose:**

This study aims to perform a bibliometric analysis of scientific research on the use of artificial intelligence (AI) in the field of Otorhinolaryngology (ORL), with a specific focus on identifying emerging AI trend topics within this discipline.

**Methods:**

A total of 498 articles on AI in ORL, published between 1982 and 2024, were retrieved from the Web of Science database. Various bibliometric techniques, including trend keyword analysis and factor analysis, were applied to analyze the data.

**Results:**

The most prolific journal was the European Archives of Oto-Rhino-Laryngology (*n* = 67). The USA (*n* = 200) and China (*n* = 61) were the most productive countries in AI-related ORL research. The most productive institutions were Harvard University / Harvard Medical School (*n* = 71). The leading authors in this field were Lechien JR. (*n* = 18) and Rameau A. (*n* = 17). The most frequently used keywords in the AI research were cochlear implant, head and neck cancer, magnetic resonance imaging (MRI), hearing loss, patient education, diagnosis, radiomics, surgery, hearing aids, laryngology ve otitis media. Recent trends in otorhinolaryngology research reflect a dynamic focus, progressing from hearing-related technologies such as hearing aids and cochlear implants in earlier years, to diagnostic innovations like audiometry, psychoacoustics, and narrow band imaging. The emphasis has recently shifted toward advanced applications of MRI, radiomics, and computed tomography (CT) for conditions such as head and neck cancer, chronic rhinosinusitis, laryngology, and otitis media. Additionally, increasing attention has been given to patient education, quality of life, and prognosis, underscoring a holistic approach to diagnosis, surgery, and treatment in otorhinolaryngology.

**Conclusion:**

AI has significantly impacted the field of ORL, especially in diagnostic imaging and therapeutic planning. With advancements in MRI and CT-based technologies, AI has proven to enhance disease detection and management. The future of AI in ORL suggests a promising path toward improving clinical decision-making, patient care, and healthcare efficiency.

**Supplementary Information:**

The online version contains supplementary material available at 10.1007/s00405-025-09272-5.

## Introduction

The field of Otorhinolaryngology (ORL) is a vital medical discipline encompassing a broad spectrum, including head and neck surgery, hearing, balance, respiration, and swallowing [1]. In recent years, the use of artificial intelligence (AI) technologies in medicine, particularly in ORL, has been rapidly increasing [2, 3]. AI has the potential to revolutionize healthcare through applications in diagnosis, treatment planning, imaging analysis, and clinical decision support systems [4]. These technologies enable ORL specialists to diagnose diseases more accurately and efficiently, develop personalized treatment approaches, and enhance the quality of healthcare services [5, 6]. The advancement of AI in this field has a wide range of impacts, extending from clinical applications to academic research [7].

The growing interest and research into AI applications in ORL underscore the significant potential of these technologies. Bibliometric analyses play a crucial role in highlighting emerging trends in fast-evolving fields like AI and ORL, offering valuable insights that guide the direction of future research and innovation. By systematically examining publications, citations, and research trends, bibliometric analyses identify research gaps, current trends, future opportunities, and the most impactful studies in the field [8, 9]. This foundation is invaluable for making strategic decisions in clinical practice and shaping the future of AI in ORL.

The aim of this study is to review the existing literature on AI in ORL, understand how these technologies are applied and developed, and identify future research directions. Specifically, we conducted analyses to identify prolific journals, institutions, and countries contributing to this field, determine key topics and research clusters through factor analysis, and examine the evolving trends in AI applications within ORL. This comprehensive bibliometric analysis provides a valuable guide for ORL specialists and researchers to explore AI’s potential applications and drive progress in this field.

## Methods

### Search strategy

A literature search was conducted using the Web of Science (WoS) database (Clarivate Analytics, Philadelphia, PA, USA) on December 1, 2024, to include studies published between 1980 and 2024. The search was limited to articles in the Otorhinolaryngology research field within the WoS database. Specific keywords and Boolean operators were used to identify AI-related articles, including terms such as “artificial intelligence”, “AI”, “ChatGPT”, “machine learning”, “deep learning”, “neural networks” combined with Boolean operators (AND, OR) to refine the results. For reproducibility of the results, the full search strategy, including all search terms, Boolean operators, filters, and limits used, is provided in Supplementary File 1. As a result of the literature search, a total of 608 publications related to AI in the field of Otorhinolaryngology were identified in the WoS database. Publications categorized as review, letter, proceeding paper, or meeting abstract etc., were excluded, and only 498 original articles categorized as “article” were included for bibliometric analysis. A flowchart illustrating the methodology is included in Supplementary File 1.

### Statistical and bibliometric analysis

Basic statistical analyses were performed using SPSS software (Version 22.0, SPSS Inc., Chicago, IL, USA, License: Hitit University). Microsoft Office Excel was used to display the temporal distribution of the published articles. Bibliometric analyses were conducted using the bibliometrix package within the R Studio tool (http://www.bibliometrix.org/), utilizing the biblioshiny interface [10]. The bibliometrix software is widely used in the scientific literature for visualizing and creating bibliometric networks and offers distinct analytical advantages compared to other software.

## Results

Almost all of the articles were published in English (*n* = 484, 97.1%), with a small number in German (*n* = 14, 2.8%). The majority of the articles were indexed in SCI-Expanded (*n* = 470, 94%), while a smaller portion was indexed in ESCI (*n* = 28, 5.6%). Among the 498 articles, the most frequently tagged research areas in the WoS index alongside Otolaryngology were Audiology and Speech-Language Pathology (70, 14.1%), Surgery (82, 16.5%), Medicine Research Experimental (48, 9.6%), Clinical Neurology (24, 4.8%), Neurosciences (18, 3.6%), Pediatrics (10, 2%), Rehabilitation (3, 0.6%), and Multidisciplinary Sciences (1, 0.2%).

**Fig. 1:**
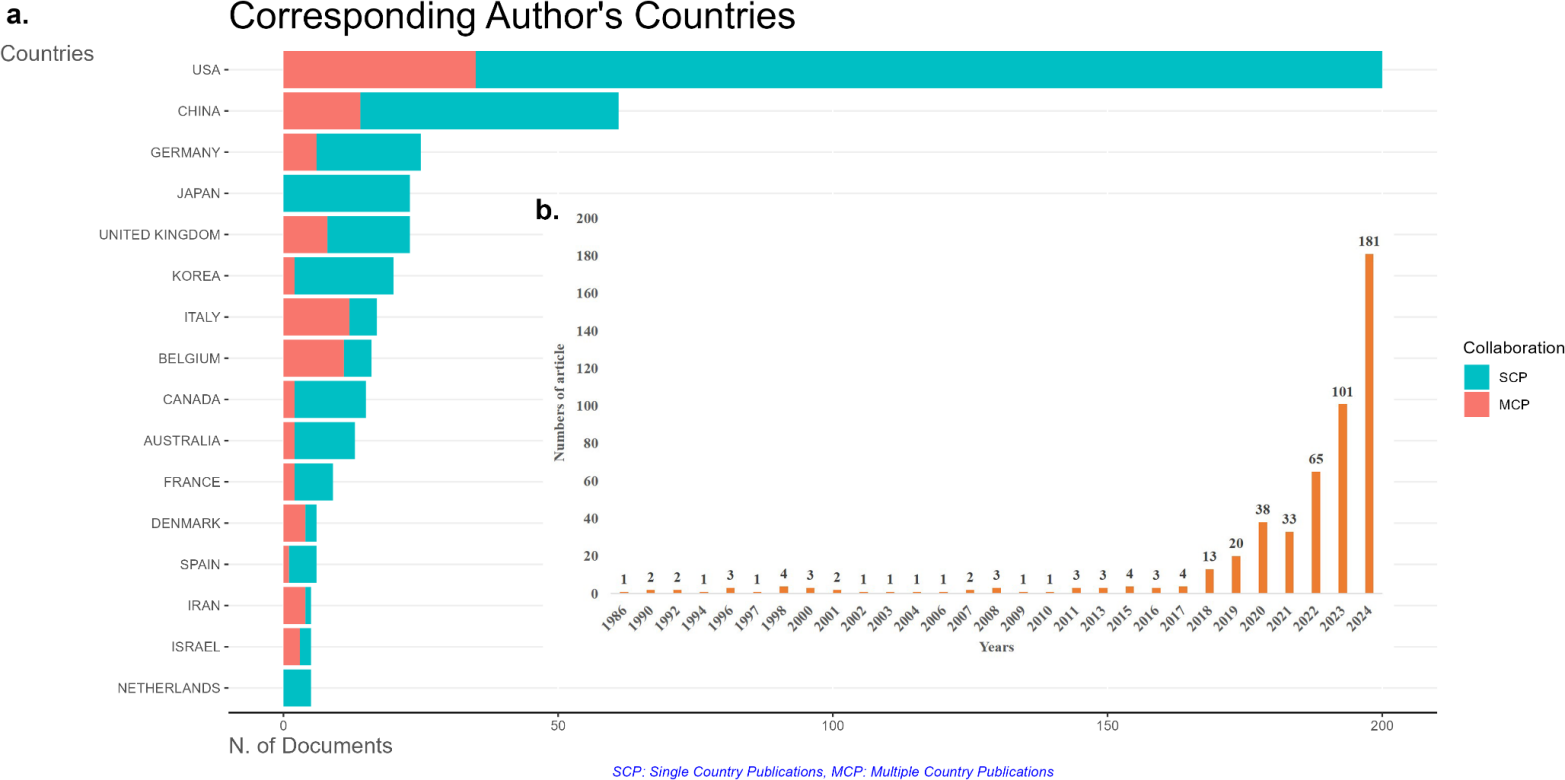
(**A**) Prolific countries with 5 or more publications on artificial intelligence, categorized by the corresponding authors’ countries. (**B**) Line graph depicting the annual variation in the number of publications on artificial intelligence in the field of otorhinolaryngology

The top five most productive countries in AI research were the USA (*n* = 200), China (*n* = 61), Germany (*n* = 25), Japan (*n* = 23), and the United Kingdom (*n* = 23). Other productive countries publishing more than 10 articles were South Korea (*n* = 20), Italy (*n* = 17), Belgium (*n* = 16), Canada (*n* = 15) and Australia (*n* = 13). Countries with five or more publications on AI, based on the corresponding authors’ affiliations, are displayed in Fig. 1a. The top five most productive journals were European Archives of Oto-Rhino-Laryngology (67), Laryngoscope (48), Otolaryngology–Head and Neck Surgery (41), Head and Neck: Journal for the Sciences and Specialties of the Head and Neck (34), and Otology & Neurotology (24). Journals publishing two or more articles on AI, along with citation analysis findings, are provided in Supplementary File 2. The top five institutions publishing the most articles were Harvard University (42), Harvard Medical School (29), the University of Toronto (26), Weill Cornell Medicine (26), and Massachusetts Eye and Ear Infirmary (25). The most prolific authors were Lechien JR. (*n* = 18), Rameau A. (17), Crowson MG. (12), Vaira LA. (12), and Chiesa-Estomba CM. (10). The table displaying the expertise and areas of focus of the top five active authors and institutions is provided in Supplementary File 3. The annual distribution of articles on AI in Otolaryngology is shown in Fig. 1b.

### Trending topic analysis on AI

**Fig. 2:**
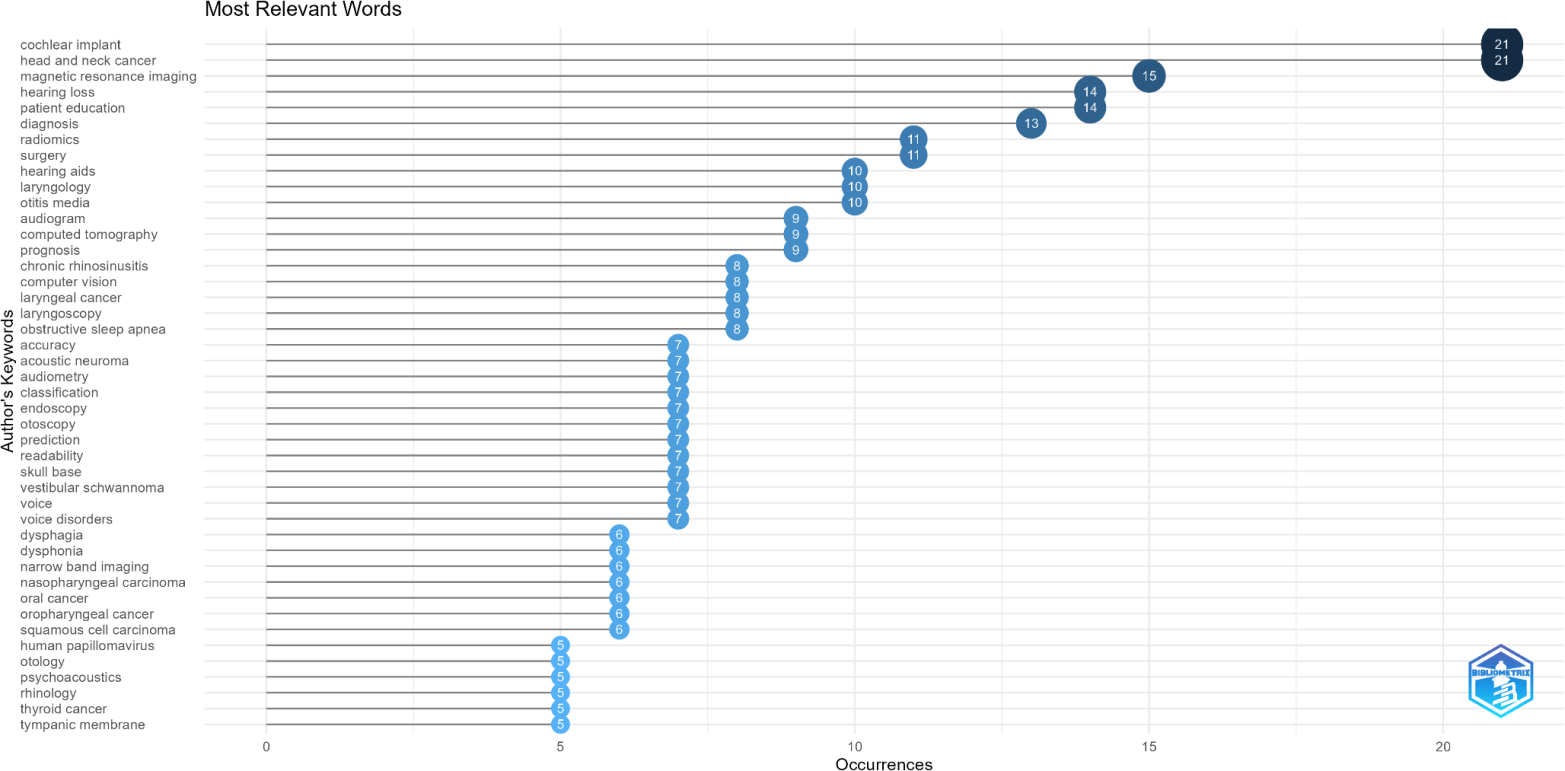
Graph illustrating the most frequently used keywords by authors in publications on artificial intelligence within the field of otorhinolaryngology

**Fig. 3:**
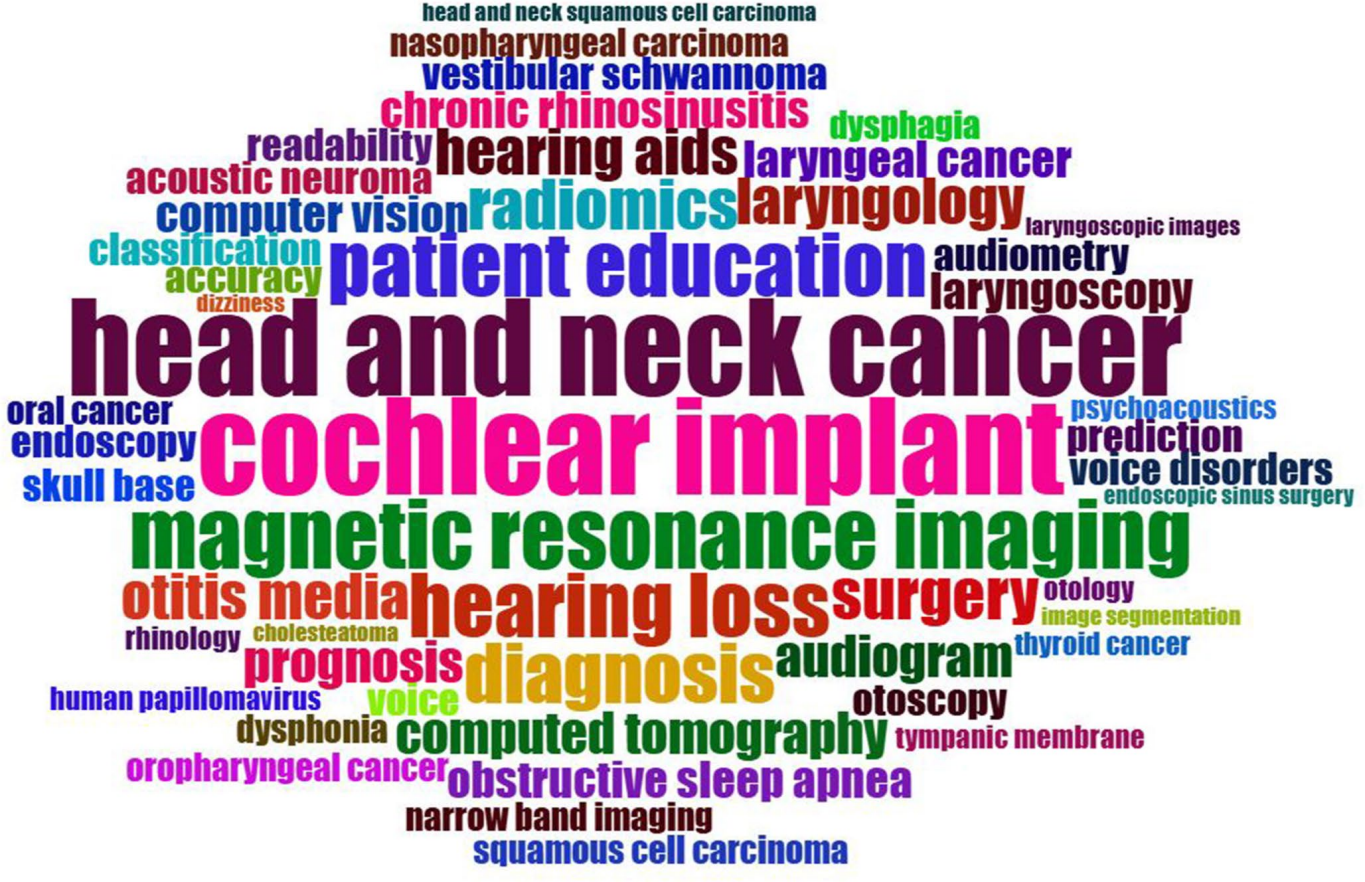
Visualization of the word cloud analysis for the top 50 keywords most frequently used by authors in the publications

A total of 1,100 unique keywords were used in the 498 articles published on AI. Keywords related to artificial intelligence (e.g., artificial intelligence, AI, ChatGPT) and research domains (e.g., otorhinolaryngology, ENT, head and neck surgery, otolaryngology) were excluded from the analysis. Additionally, synonymous terms such as “computed tomography, CT,” “laryngeal cancer, laryngeal carcinoma,” and “cochlear implant, cochlear implants” were consolidated prior to the analysis. The top 44 most frequently used author keywords in conjunction with AI, appearing in five or more articles, are presented in Fig. 2. In AI research within the ORL field, keywords that were used in over 10 articles included cochlear implant, head and neck cancer, magnetic resonance imaging (MRI), hearing loss, patient education, diagnosis, radiomics, surgery, hearing aids, laryngology, and otitis media (Fig. 2). A word cloud visualization depicting the proportional density of the top 50 most commonly used keywords is shown in Fig. 3.

**Fig. 4:**
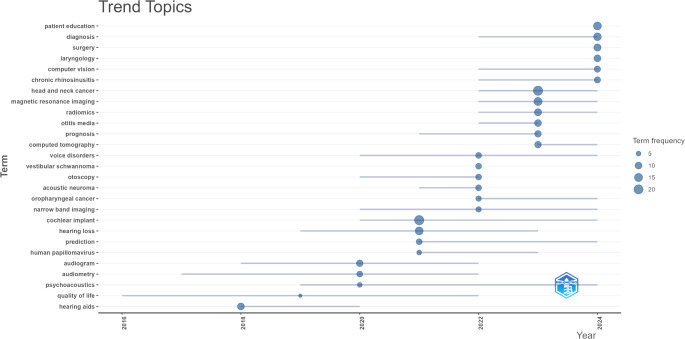
Graph displaying the trend of keywords over the years in publications on artificial intelligence within the field of otorhinolaryngology. **Footnote**: Horizontal lines indicate the years in which the keywords were used, and the size of the circles represents their frequency of use

A trend analysis of author keywords was performed to explore temporal shifts. The findings based on a minimum co-occurrence of four different articles are presented in Fig. 4. Based on the trend keyword analysis findings, hearing aids emerged as a trending topic in 2018, while quality of life gained prominence in 2019. Topics such as psychoacoustics, audiometry, and audiogram were identified as trends in 2020, followed by human papillomavirus, hearing loss, and cochlear implant in 2021. In 2022, narrow band imaging, voice disorders, vestibular schwannoma, otoscopy, acoustic neuroma, and oropharyngeal cancer were highlighted as trending topics. In 2023, the focus shifted to topics such as prognosis, computed tomography (CT), radiomics, otitis media, MRI, and head and neck cancer. By 2024, chronic rhinosinusitis, computer vision, laryngology, surgery, diagnosis, and patient education emerged as the prominent trending topics.

**Fig. 5:**
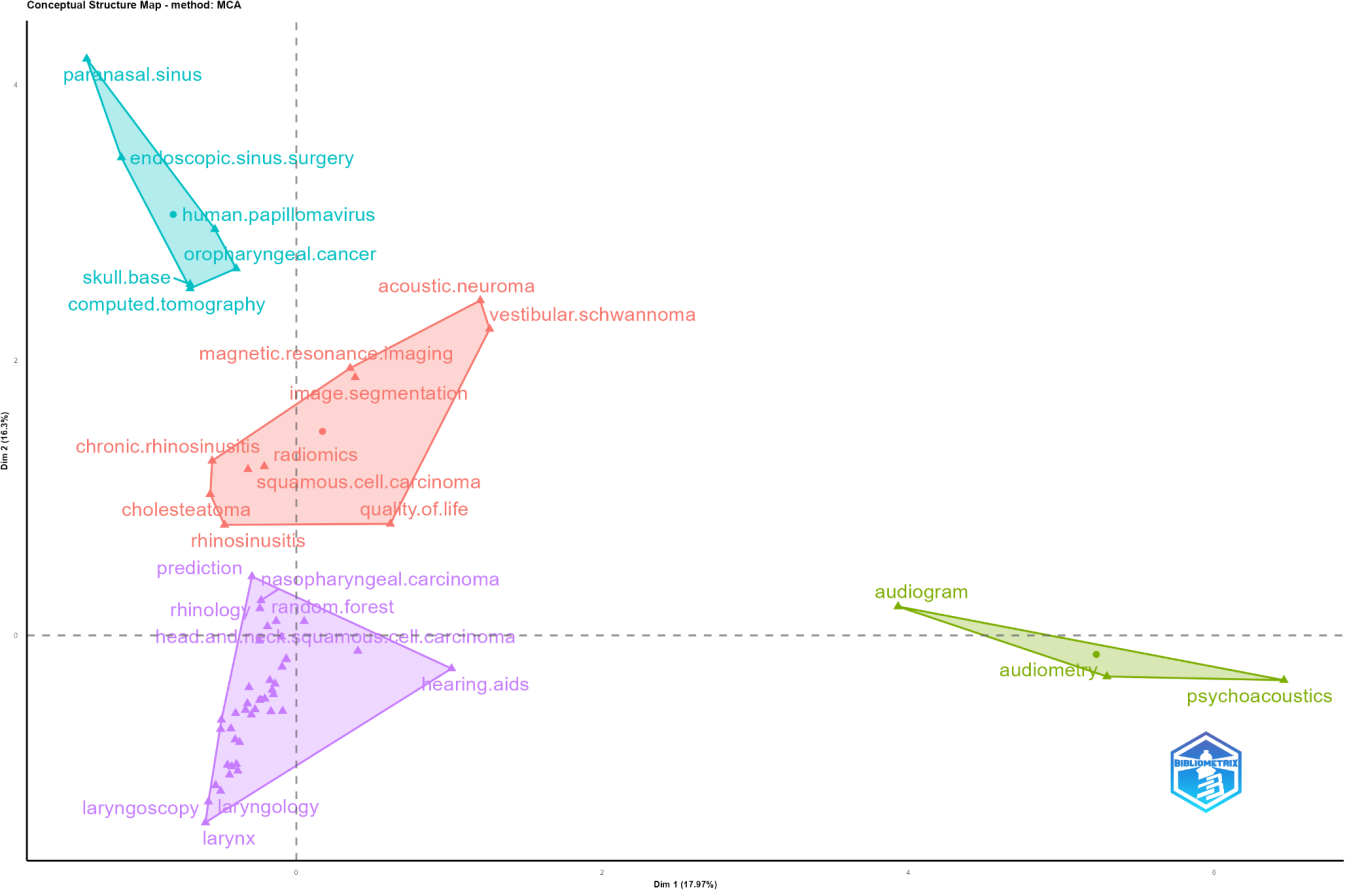
Factor analysis conducted using Multiple Correspondence Analysis. **Footnote**: The center of the map represents the core of the research topic, highlighting the primary subjects. Each color denotes a different sub-dimension

The results of the factor analysis categorized the AI literature in ORL into four primary clusters (Fig. 5), providing a deeper understanding of the conceptual framework in this field. The most comprehensive cluster at the center reflects AI applications in the primary topics of ORL. The second cluster, closest to the center, primarily represents AI applications based on visual data analysis, particularly MRI-based imaging methods, while the third cluster reflects AI applications utilizing CT imaging. The fourth cluster emphasizes the role of AI in hearing-related studies. All keywords within these clusters are provided in Supplementary File 4.

## Discussion

An analysis of the yearly distribution of AI publications reveals a rapid increase starting in 2018, peaking in 2024. This trend highlights the growing significance of artificial intelligence in the field of otorhinolaryngology and suggests that this technology will become even more impactful in the coming years. The rapid growth in the number of studies, particularly in recent years, strongly indicates that AI will hold a prominent role in areas such as diagnosis, treatment, surgical interventions, and patient monitoring.

The geographical distribution of AI research in ORL reveals that the United States leads significantly, followed by China, Germany, Japan, and the United Kingdom. This distribution is likely influenced by several factors, including the economic strength and developmental status of these countries, which enable greater investment in research and development (R&D). Nations like the USA and China allocate substantial funding for scientific research, fostering innovation in cutting-edge fields such as AI. Additionally, these countries have a high density of advanced universities and research laboratories, which serve as hubs for technological development. The availability and accessibility of AI technologies in these regions also play a pivotal role, as do their well-established collaborations between academic and industrial sectors. Previous studies have highlighted that the infrastructure and resources in these nations significantly contribute to their higher productivity in scientific research [8, 9].

The most frequently used keyword in the ORL field is cochlear implant (CI). AI offers various solutions to enhance clinical applications and optimize user outcomes in the context of CI [11]. For instance, patients with cochlear implants often struggle with communication in noisy environments. Noise masks speech signals, making it difficult for patients to perceive and understand speech and leading to challenges in selective hearing in environments with multiple speakers. It has been determined that AI can improve speech intelligibility for cochlear implant users in noisy settings through noise reduction algorithms and speech enhancement methods [12, 13]. In cochlear implant surgery, numerous factors influence the postoperative effectiveness of the device, including the patient’s demographic data, cochlear nerve function, hearing test results, and cognitive perception. AI has developed models that analyze individual patient data to predict postoperative success [14]. These algorithms predict potential outcomes by utilizing data from previous patients. AI has been reported to assist in creating personalized treatment plans by predicting surgical success [15]. Additionally, AI-based efficiency tools enable remote device adjustments and accelerate programming processes [16, 17]. Robotic-assisted techniques and electrode placement strategies optimized with AI have also been shown to make surgical procedures safer and more effective [18].

The second most frequently researched topic in AI is Head and Neck Cancer. Head and neck cancers are of significant importance in ORL practice due to their high mortality and morbidity rates. In this group of diseases, the early diagnosis of tumors, accurate staging, and the development of effective treatment plans play a critical role in patient survival. AI-based machine learning algorithms enhance treatment processes by predicting conditions such as lymph node metastasis and delays in postoperative adjuvant radiotherapy [19, 20]. Prognostic models derived from electronic health records and radiomic data have been shown to provide more precise outcomes in predicting patient survival compared to traditional methods [21]. Moreover, AI-driven pathomic models provide critical insights into tumor biology and gene expression, enabling prognosis prediction [22]. AI-supported decision support systems and chatbots improve patient counseling during diagnosis and treatment [2], while also being effective in surgical planning and the management of postoperative complications [23]. In rehabilitation processes, such as evaluating voice and swallowing functions, AI offers personalized approaches to improve patients’ quality of life [24]. In this context, the integration of AI into clinical applications for head and neck cancers accelerates diagnostic and treatment processes while enabling the development of more personalized and effective treatment plans.

The third most common application is the processing of MRI images using AI. Due to the complex anatomical structures in the head and neck region, MRI imaging provides detailed visualization, frequently used for detecting tumoral structures. However, challenges such as time-consuming analyses, the need for expert evaluation, and the complex nature of images persist. AI enhances the effectiveness of MRI imaging in ORL by addressing these challenges. Through radiomics and deep learning algorithms, AI can automatically analyze MRI data, predicting tumor size, malignancy risk, lymph node metastasis, bone invasion, and postoperative complications [25, 26]. For instance, it aids in assessing the malignancy risk of lesions in inverted papillomas, enabling accurate diagnosis and treatment planning [27]. AI-based models have achieved significant success in tasks such as tumor segmentation in vestibular schwannomas [28], differential diagnosis of parotid tumors [29], and predicting radiation-induced complications in nasopharyngeal cancer [30]. Additionally, by comparing CT and MRI in cholesteatoma diagnosis, AI demonstrates its potential to improve imaging accuracy and play a vital role in clinical decision support systems [31]. These methods not only enhance diagnostic accuracy but also personalize treatment processes and accelerate clinical decision-making.

Hearing loss has also been a frequently researched topic using AI. Early diagnosis and intervention are crucial for patients with hearing loss, including preventable childhood cases that may delay language development and adult cases linked to depression and cognitive decline [32]. Predicting hearing loss in advance offers opportunities for early treatment, improving the effectiveness of hearing aid use, and preventing exposure-related losses in individuals working in noisy environments [33]. AI has developed early warning systems across a wide spectrum, from industrial noise-exposed workers [34] to preventable hearing loss in children [35]. Additionally, in challenging conditions such as sudden sensorineural hearing loss, AI has emerged as a powerful tool for predicting disease prognosis through big data analysis and machine learning algorithms [36, 37].

AI has also been frequently studied in the context of patient education. Patients often turn to internet searches, social media platforms, or general health websites to access information. However, these sources pose significant risks regarding accuracy and reliability. Incorrect, incomplete, or unverified information may increase patients’ anxiety, lead them to inappropriate treatments, or result in unnecessary medical interventions. The complexity of medical terminology often makes it difficult for patients to comprehend accurate information and incorporate it into their healthcare decisions. Here, AI offers an effective solution for improving patient education. AI-powered chatbots like ChatGPT can answer patient questions, simplify medical information, and provide personalized insights [38]. For example, in otolaryngology, AI has facilitated patient education by making information about conditions such as anosmia, tracheotomy, and laryngopharyngeal reflux more understandable and accessible [39, 40, 41]. Additionally, AI has been noted to simplify complex information about surgical procedures, enhancing patient understanding [42].

Apart from these keywords, numerous other terms in the field of otorhinolaryngology (ORL) stand out in AI-related studies (Figs. 2 and 3). This suggests that AI will play an increasingly significant role in ORL in the future. The rapid growth of AI technologies in the ORL field in recent years has made substantial contributions to this discipline. Our bibliometric analysis revealed that trending keywords have shifted over the years in AI-related ORL research. While specific topics like hearing aids in 2018 and quality of life in 2019 were prominent, from 2020 onward, there has been a focus on more technical areas, such as audiometry, psychoacoustics, and audiograms. Recent years have highlighted advanced technologies like MRI and radiomic analysis, along with applications in chronic rhinosinusitis, laryngology, and surgical procedures. These trends demonstrate that AI is transforming not only diagnostic and treatment processes but also broader clinical applications, such as patient education and prognosis prediction. The observed shift in research focus from hearing-related technologies to diagnostic imaging, patient education, and holistic patient management may reflect advancements in imaging technologies and the growing recognition of AI’s potential beyond isolated therapeutic areas. While hearing-related technologies remain critical, the expansion of AI applications to encompass broader clinical contexts, such as radiomics and educational tools, aligns with an evolving emphasis on personalized and comprehensive care models in medicine. These shifts are likely driven by the increasing availability of AI tools capable of handling complex data types, such as imaging and clinical records, alongside a heightened focus on patient-centered care.

In 2023, some of the priority trends in ORL included the potential applications of AI in MRI and head and neck cancer treatment. Another significant trend was radiomics. A study focusing on AI and radiomic analyses examined CT radiomic models developed to non-invasively predict granzyme A in head and neck squamous cell carcinoma and machine learning models for external auditory canal CT scans to evaluate the feasibility of endoscopic ear surgery. These technologies demonstrate the potential to enhance diagnostic accuracy and improve surgical processes [43]. Research on AI and clinical decision support systems in pediatric otitis media treatment highlights the efficacy of deep learning approaches in diagnosing atelectasis and attic retraction pockets using otoscopic images. Quality improvement efforts in clinical decision-making processes for pediatric otitis media treatment were also emphasized [44, 45]. Studies linking AI with prognosis illustrate the capabilities of AI-based deep learning models to predict treatment outcomes from nasal polyp histology Sect. [46] and forecast hearing prognosis after intact canal wall mastoidectomy and tympanoplasty. These advances improve surgical treatment processes and patient outcome predictions [47]. Furthermore, research on AI and CT underscores the potential of deep learning algorithms in differentiating eosinophilic from non-eosinophilic chronic rhinosinusitis in preoperative CT images [48] and detecting anatomical landmarks in temporal bone CT scans, enhancing diagnostic accuracy and surgical planning [49].

In 2024, AI applications focusing on patient education emerged as a significant trend in ORL. Chronic rhinosinusitis became another focal point, with AI playing a role in diagnosis [50], predicting postoperative outcomes [51], and patient education [52]. AI-based tools have been explored for their potential in assisting diagnosis and treatment across various otolaryngology fields, including head and neck cancers [2, 19, 22, 24], ENT diseases [5, 6, 7], pediatric otitis media [53], laryngopharyngeal cancer [3], cervical lymphadenopathy [54], and vocal cord leukoplakia [55]. These technologies offer accuracy, reliability, and innovative approaches compared to traditional methods. Studies linking AI and surgery comprehensively examined the roles of ChatGPT and other AI-based tools in otolaryngology and head-neck surgery, focusing on diagnosis, treatment, patient education, preoperative counseling, postoperative monitoring, clinical decision support systems, and surgical processes. These tools were found to enhance accuracy, reliability, readability, and clinical applications [5, 6, 56, 57]. Research on AI and computer vision demonstrated their capacity to bridge gaps between clinical needs and AI capabilities, improving diagnostic accuracy and surgical workflows [7]. Additionally, AI-driven studies in laryngology highlighted ChatGPT’s potential in answering patient questions, improving educational materials, supporting clinical decisions, addressing ethical challenges, ensuring guideline compliance, and creating evaluative educational materials [5, 6, 7].

The identified research trends underscore AI’s transformative potential in clinical decision-making, patient care, and healthcare efficiency within ORL. For example, advancements in diagnostic imaging, such as MRI and CT-based radiomics, have significantly enhanced diagnostic accuracy and surgical planning. Patient education tools, including AI-driven chatbots, improve patient understanding and engagement, leading to better compliance with treatment plans. Furthermore, AI’s role in prognostic modeling enables more precise predictions of treatment outcomes, facilitating personalized care pathways. Collectively, these advancements contribute to streamlined workflows, reduced clinical errors, and improved healthcare delivery, underscoring the far-reaching benefits of AI integration in ORL.

The trends identified in ORL research largely parallel broader developments in AI applications within the medical domain. Across various specialties, there has been a notable transition from task-specific applications, such as predictive models for isolated conditions, to integrative approaches that leverage AI for diagnostic imaging, patient education, and multidisciplinary management. For example, radiomics and deep learning, which are now prevalent in ORL, have also seen widespread adoption in oncology and neurology [58]. Similarly, patient education tools, such as AI-driven chatbots, are gaining traction in fields like primary care and pediatrics [59, 60]. These parallels suggest that ORL is keeping pace with advancements in the broader medical community, demonstrating how interdisciplinary innovations can translate into specialty-specific benefits.

The factor analysis results provided valuable insights into the conceptual framework of AI research in ORL by categorizing the literature into four main clusters (Fig. 5). The first and most comprehensive cluster represents the primary applications of AI in ORL, focusing on topics such as cochlear implants, head and neck cancer, hearing loss, and patient education. This cluster emphasizes AI’s role in diagnosing, treating, and managing various ORL pathologies, reflecting its wide-ranging clinical applications. The second cluster highlights AI applications in visual data analysis, particularly MRI-based imaging methods. This includes advanced topics like radiomics, chronic rhinosinusitis, and the segmentation of acoustic neuroma and vestibular schwannoma, showcasing how AI enhances diagnostic accuracy and surgical planning. The third cluster is centered around CT imaging and its applications in conditions such as oropharyngeal cancer, skull base pathologies, and paranasal sinus diseases, illustrating AI’s potential in improving imaging-based diagnosis and treatment strategies. The fourth cluster focuses on hearing-related AI applications, including audiograms, audiometry, and psychoacoustics. These findings underscore the diverse scope of AI in ORL, spanning from imaging innovations to patient-centered applications, and highlight its transformative potential across multiple domains in the field.

The clustering of research topics in ORL-AI highlights opportunities for collaboration and interdisciplinary innovation. For instance, the integration of imaging-based clusters (MRI and CT applications) with patient-centered clusters (education and hearing loss management) could lead to the development of comprehensive decision-support tools. These synergies emphasize the potential for collaboration between radiologists, surgeons, and data scientists. Additionally, clusters such as radiomics and psychoacoustics suggest untapped research areas, including the role of AI in bridging auditory diagnostics with imaging technologies. Identifying these intersections can inform targeted research initiatives, fostering advancements that benefit multiple disciplines.

This study has certain limitations. First, as the data were obtained solely from a specific database (WoS), studies available in other databases were not included in the analysis, which might limit the scope of the literature. Nonetheless, WoS was chosen as a reliable data source for scientific bibliometric studies due to its inclusion of high-quality, peer-reviewed publications, its broad coverage, and its provision of detailed citation analyses [8, 9]. Second, as this is a cross-sectional observational study, the analysis is based on data available at a specific point in time. Future indexing and newly published studies may alter these findings. Additionally, bibliometric analyses, while valuable for identifying trends and patterns, may benefit from complementary qualitative approaches to provide deeper insights into the content and implications of the research. Despite these limitations, the study offers a comprehensive overview of AI research in ORL, highlighting significant trends and areas for future exploration.

## Conclusion

This study demonstrates that the use of artificial intelligence in the field of otorhinolaryngology (ORL) is rapidly increasing, with significant potential across various clinical stages, including diagnosis, treatment, surgical interventions, patient monitoring, and education. AI is transforming ORL practice, particularly through its accuracy and efficiency in imaging techniques and psychoacoustic analyses. Recent research indicates that AI not only enhances existing diagnostic and treatment processes but also plays an active role in determining disease prognosis and improving patient education. In this context, the future of AI in ORL appears to offer a promising roadmap for increasing the efficiency of healthcare services and improving patient management. Future research should focus on further exploring emerging trends, such as AI applications in patient education, prognostic modeling, and interdisciplinary synergies, to maximize the potential of these technologies in ORL.

## Electronic supplementary material

Below is the link to the electronic supplementary material.

Supplementary Material 1

Supplementary Material 2

Supplementary Material 3

Supplementary Material 4

## Data Availability

Data can be obtained by contacting the corresponding author.
