# Supplementary material for: Artificial intelligence in otorhinolaryngology: current trends and application areas
Source: Eur Arch Otorhinolaryngol. 2025 Feb 17;282(5):2697–707. doi: 10.1007/s00405-025-09272-5 (PMC12055906; doi:10.1007/s00405-025-09272-5)
Supplement: Supplementary file 1 — Supplementary Material 1 [file 405_2025_9272_MOESM1_ESM.docx]

**Table.** Reproducibility codes for researchers

| **Date** | December 1, 2024 (It should be noted that searches conducted on different dates may yield a different number of articles due to newly indexed studies added later) |
| --- | --- |
| **WoS codes** | ((WC=(otorhinolaryngology) AND TI=(“artificial intelligence”)) OR  (WC=(otorhinolaryngology) AND TI=(AI) AND TS=(“artificial intelligence”)) OR  (WC=(otorhinolaryngology) AND TI=(machine learning)) OR  (WC=(otorhinolaryngology) AND TI=("deep learning")) OR  (WC=(otorhinolaryngology) AND TI=("neural network*")) OR  (WC=(otorhinolaryngology) AND TI=(“natural language proces*”)) OR  (WC=(otorhinolaryngology) AND TI=(supervised learning)) OR  (WC=(otorhinolaryngology) AND TI=(unsupervised Learning)) OR  (WC=(otorhinolaryngology) AND TI=(ChatGPT)) OR  (WC=(otorhinolaryngology) AND TI=(Chat-GPT)) OR  (WC=(otorhinolaryngology) AND TI=("Data Mining")) OR  (WC=(otorhinolaryngology) AND TI=("Big data")) OR  (WC=(otorhinolaryngology) AND TI=("image processing")) OR  (WC=(otorhinolaryngology) AND TI=("Clinical Decision Making")) OR  (WC=(otorhinolaryngology) AND TI=("Clinical Decision support")) OR  (WC=(otorhinolaryngology) AND TI=("Decision Support System*"))) |
| **Refined By:** | Document types: Article |

The use of an asterisk allows the inclusion of articles with terms such as ‘neural networks’ in the title in addition to articles with ‘neural network’ in the title.

**Records identified through Web of Science searching in the Otorhinolaryngology research field (n=608)**

**Publication types other than articles**

**Excluded (n=110)**

- Review Article
- Editorial Material
- Letter
- Proceeding Paper
- Correction
- Book Chapters
- Meeting Abstract
- News Item

**Studies included in bibliometric analysis (n=498)**

**Figure 1.** Flowchart
