# Supplementary material for: Artificial intelligence in otorhinolaryngology: current trends and application areas
Source: Eur Arch Otorhinolaryngol. 2025 Feb 17;282(5):2697–707. doi: 10.1007/s00405-025-09272-5 (PMC12055906; doi:10.1007/s00405-025-09272-5)
Supplement: Supplementary file 2 — Supplementary Material 2 [file 405_2025_9272_MOESM2_ESM.docx]

**Table:** Journals publishing 2 or more articles on artificial intelligence in Otolaryngology

| **Journals** | **NA** | **TC** | **ACPA** | **h-index** | **m-index** | **PY start** |
| --- | --- | --- | --- | --- | --- | --- |
| European Archives of Oto-Rhino-Laryngology | 67 | 460 | 6.87 | 11 | 0.58 | 2006 |
| Laryngoscope | 48 | 608 | 12.67 | 15 | 0.65 | 2002 |
| Otolaryngology-Head and Neck Surgery | 41 | 253 | 6.17 | 10 | 0.37 | 1998 |
| Head and Neck-Journal for the Sciences and Specialties of The Head and Neck | 34 | 266 | 7.82 | 10 | 0.71 | 2011 |
| Otology & Neurotology | 24 | 157 | 6.54 | 8 | 0.44 | 2007 |
| American Journal of Otolaryngology | 21 | 125 | 5.95 | 7 | 1.75 | 2021 |
| International Forum of Allergy & Rhinology | 18 | 214 | 11.89 | 7 | 1.17 | 2019 |
| Ear And Hearing | 15 | 297 | 19.80 | 9 | 0.53 | 2008 |
| Journal of Voice | 14 | 369 | 26.36 | 9 | 0.53 | 2008 |
| Otolaryngologic Clinics of North America | 14 | 4 | 0.29 | 1 | 0.33 | 2022 |
| Acta Oto-Laryngologica | 12 | 94 | 7.83 | 6 | 0.17 | 1990 |
| Laryngoscope Investigative Otolaryngology | 12 | 95 | 7.92 | 6 | 1.00 | 2019 |
| Trends in Hearing | 12 | 115 | 9.58 | 6 | 0.86 | 2018 |
| Clinical Otolaryngology | 12 | 114 | 9.50 | 5 | 0.63 | 2017 |
| International Journal of Pediatric Otorhinolaryngology | 10 | 28 | 2.80 | 3 | 0.33 | 2016 |
| Journal of Laryngology and Otology | 9 | 93 | 10.33 | 5 | 0.83 | 2019 |
| Oto Open | 9 | 55 | 6.11 | 3 | 1.50 | 2023 |
| Laryngo-Rhino-Otologie | 9 | 19 | 2.11 | 2 | 0.06 | 1992 |
| Hearing Research | 8 | 162 | 20.25 | 6 | 0.67 | 2016 |
| American Journal of Audiology | 8 | 54 | 6.75 | 5 | 0.71 | 2018 |
| Dysphagia | 7 | 57 | 8.14 | 4 | 0.40 | 2015 |
| Ent-Ear Nose & Throat Journal | 7 | 5 | 0.71 | 1 | 0.50 | 2023 |
| JAMA Otolaryngology-Head & Neck Surgery | 6 | 137 | 22.83 | 5 | 0.50 | 2015 |
| Annals of Otology Rhinology And Laryngology | 6 | 40 | 6.67 | 4 | 0.12 | 1992 |
| Journal of Craniovertebral Junction and Spine | 6 | 55 | 9.17 | 4 | 0.80 | 2020 |
| Auris Nasus Larynx | 6 | 24 | 4.00 | 2 | 0.67 | 2022 |
| International Journal of Audiology | 5 | 36 | 7.20 | 3 | 0.50 | 2019 |
| HNO | 5 | 11 | 2.20 | 2 | 0.33 | 2019 |
| Clinical and Experimental Otorhinolaryngology | 4 | 45 | 11.25 | 3 | 0.60 | 2020 |
| JARO-Journal of the Association for Research in Otolaryngology | 4 | 75 | 18.75 | 2 | 0.08 | 2000 |
| Journal of International Advanced Otology | 4 | 22 | 5.50 | 2 | 0.22 | 2016 |
| Current Opinion in Otolaryngology & Head and Neck Surgery | 4 | 1 | 0.25 | 1 | 1.00 | 2024 |
| Audiology and Neurotology | 3 | 15 | 5.00 | 2 | 0.40 | 2020 |
| Cochlear Implants International | 3 | 23 | 7.67 | 2 | 0.40 | 2020 |
| Scandinavian Audiology | 3 | 12 | 4.00 | 2 | 0.07 | 1996 |
| World Journal of Otorhinolaryngology-Head & Neck Surgery | 3 | 2 | 0.67 | 1 | 0.50 | 2023 |
| Australian Journal of Otolaryngology | 2 | 5 | 2.50 | 1 | 0.25 | 2021 |

ACPA: Average citations per article

NA: Number of article

TC: Total Citation

PY: Publication Year
