# Supplementary material for: Artificial intelligence in otorhinolaryngology: current trends and application areas
Source: Eur Arch Otorhinolaryngol. 2025 Feb 17;282(5):2697–707. doi: 10.1007/s00405-025-09272-5 (PMC12055906; doi:10.1007/s00405-025-09272-5)
Supplement: Supplementary file 3 — Supplementary Material 3 [file 405_2025_9272_MOESM3_ESM.docx]

**Table.** Expertise and areas of focus of the active authors and institutions

|  | **Area of Expertise** | **Focus Areas** |
| --- | --- | --- |
| **Active authors** | - Head-Neck Surgery, Head-Neck Oncology, - Laryngology, Laryngeal Imaging, Laryngoscopic, Laryngopharyngeal Reflux, - Tracheotomy, - Oromaxillofacial Surgery, - Obstructive Sleep Apnea, - Otolaryngology, Pediatric Otology, - Thyroid Cancer, - Rhinology, - Oral Oncology, - Reconstructive Surgery - Voice - Cochlear implant - Chronic rhinosinusitis with nasal polyps (CRSwNP) - Sialendoscopy, | - Integration of AI in head and neck surgery - Accuracy, validity, and reliability of AI performance evaluation tools in laryngology and head and neck surgery - Comparison of the quality of information provided by AI chatbots (ChatGPT4 vs Claude2) in the context of reconstructive surgery for head and neck cancer - AI in laryngopharyngeal reflux and tracheotomy - The accuracy of information provided by ChatGPT regarding head and neck and oromaxillofacial surgery. - Role of AI in managing obstructive sleep apnea - Introduction of machine learning and generative AI to otolaryngology specialists - Predicting depression onset from hearing loss using machine learning algorithms - Thyroid cancer risk stratification using AI - ChatGPT-4's effectiveness in patient education for rhinology - Automated vocal fold polyp classification - Progression of oral lesions to malignancy - Gender identification from voice data - Limitations in voice AI research - Opportunities and challenges in cochlear implantation - Analysis of FDA (Food and Drug Administration) adverse event reports for cochlear implants with AI - The reliability of ChatGPT in synthesizing treatment guidelines for biological CRSwNP treatment. - Use of ChatGPT as a clinical decision support tool for sialendoscopy |
| **Active**  **institutions** | - Head and neck cancer, Head and neck surgery, - Pediatric otitis media, Otitis media, - Rhinology, - Endoscopic thyroid surgery, Parathyroid recognition, - Voice - Laryngology, Laryngopharyngeal reflux, - Broncho-Esophagology, - Sleep surgery, - Otolaryngology, - Hearing loss, - Oral lesions, Oral Cancer Surgery - Oropharyngeal squamous cell carcinoma, Oropharyngeal Cancer, - Cochlea, Cochlear Implantation, - Neural degeneration, - Glottic angle, | - AI and machine learning applications in predicting surgical outcomes in head and neck cancer. - Validation of deep learning models for diagnosing pediatric middle ear infections. - Comparison of AI-driven otoscopy with clinician assessments for otitis media. - AI applications in rhinology and thyroid surgery, expanding use for early parathyroid issue detection and improving ischemia management. - Advancements in voice care, including AI-driven data collection and gender identification for voice-related healthcare, despite ongoing research challenges. - Role of artificial intelligence in laryngology, broncho-esophagology, and sleep surgery - ChatGPT and natural language data mining enhancing medical education in otolaryngology. - Machine learning for predicting depression in individuals with hearing loss, analyzing oral lesion progression, and determining HPV status and survival rates in oropharyngeal cancer using 3D models. - AI’s role in auditory health, analyzing neural degeneration in cochleas. - Machine learning in surgical contexts, predicting outcomes such as the anterior glottic angle in vocal fold immobility, hospital stays for oral cancer surgery, and unplanned visits for head and neck cancer patients. - These innovations highlight the transformative potential of AI in optimizing workflows, improving diagnostic accuracy, and advancing patient outcomes in otolaryngology. |
