# Supplementary material for: Artificial intelligence in otorhinolaryngology: current trends and application areas
Source: Eur Arch Otorhinolaryngol. 2025 Feb 17;282(5):2697–707. doi: 10.1007/s00405-025-09272-5 (PMC12055906; doi:10.1007/s00405-025-09272-5)
Supplement: Supplementary file 4 — Supplementary Material 4 [file 405_2025_9272_MOESM4_ESM.docx]

**Table.** Key words in the clusters obtained as a result of factor analysis

| **Keywords** | **Cluster** |
| --- | --- |
| cochlear implant | 1 |
| head and neck cancer | 1 |
| hearing loss | 1 |
| patient education | 1 |
| diagnosis | 1 |
| surgery | 1 |
| hearing aids | 1 |
| laryngology | 1 |
| otitis media | 1 |
| prognosis | 1 |
| computer vision | 1 |
| laryngeal cancer | 1 |
| laryngoscopy | 1 |
| obstructive sleep apnea | 1 |
| accuracy | 1 |
| classification | 1 |
| endoscopy | 1 |
| otoscopy | 1 |
| prediction | 1 |
| readability | 1 |
| voice | 1 |
| voice disorders | 1 |
| dysphagia | 1 |
| dysphonia | 1 |
| narrow band imaging | 1 |
| nasopharyngeal carcinoma | 1 |
| oral cancer | 1 |
| otology | 1 |
| rhinology | 1 |
| thyroid cancer | 1 |
| tympanic membrane | 1 |
| dizziness | 1 |
| head and neck squamous cell carcinoma | 1 |
| laryngoscopic images | 1 |
| larynx | 1 |
| letters of recommendation | 1 |
| meniere s disease | 1 |
| outcome prediction | 1 |
| performance | 1 |
| random forest | 1 |
| sinusitis | 1 |
| thyroidectomy | 1 |
| magnetic resonance imaging | 2 |
| radiomics | 2 |
| chronic rhinosinusitis | 2 |
| acoustic neuroma | 2 |
| vestibular schwannoma | 2 |
| squamous cell carcinoma | 2 |
| cholesteatoma | 2 |
| image segmentation | 2 |
| quality of life | 2 |
| rhinosinusitis | 2 |
| computed tomography | 3 |
| skull base | 3 |
| oropharyngeal cancer | 3 |
| human papillomavirus | 3 |
| endoscopic sinus surgery | 3 |
| paranasal sinus | 3 |
| audiogram | 4 |
| audiometry | 4 |
| psychoacoustics | 4 |
